# Supplementary material for: The Construction and Exploration of a Comprehensive MicroRNA Centered Regulatory Network in Foxtail Millet (Setaria italica L.)
Source: Front Plant Sci. 2022 May 6;13:848474. doi: 10.3389/fpls.2022.848474 (PMC9121102; doi:10.3389/fpls.2022.848474)
Supplement: Supplementary file 4 [file Data_Sheet_4.zip › Sit-miR398b_Seita.4G288600.1_1068_TPlot.pdf]

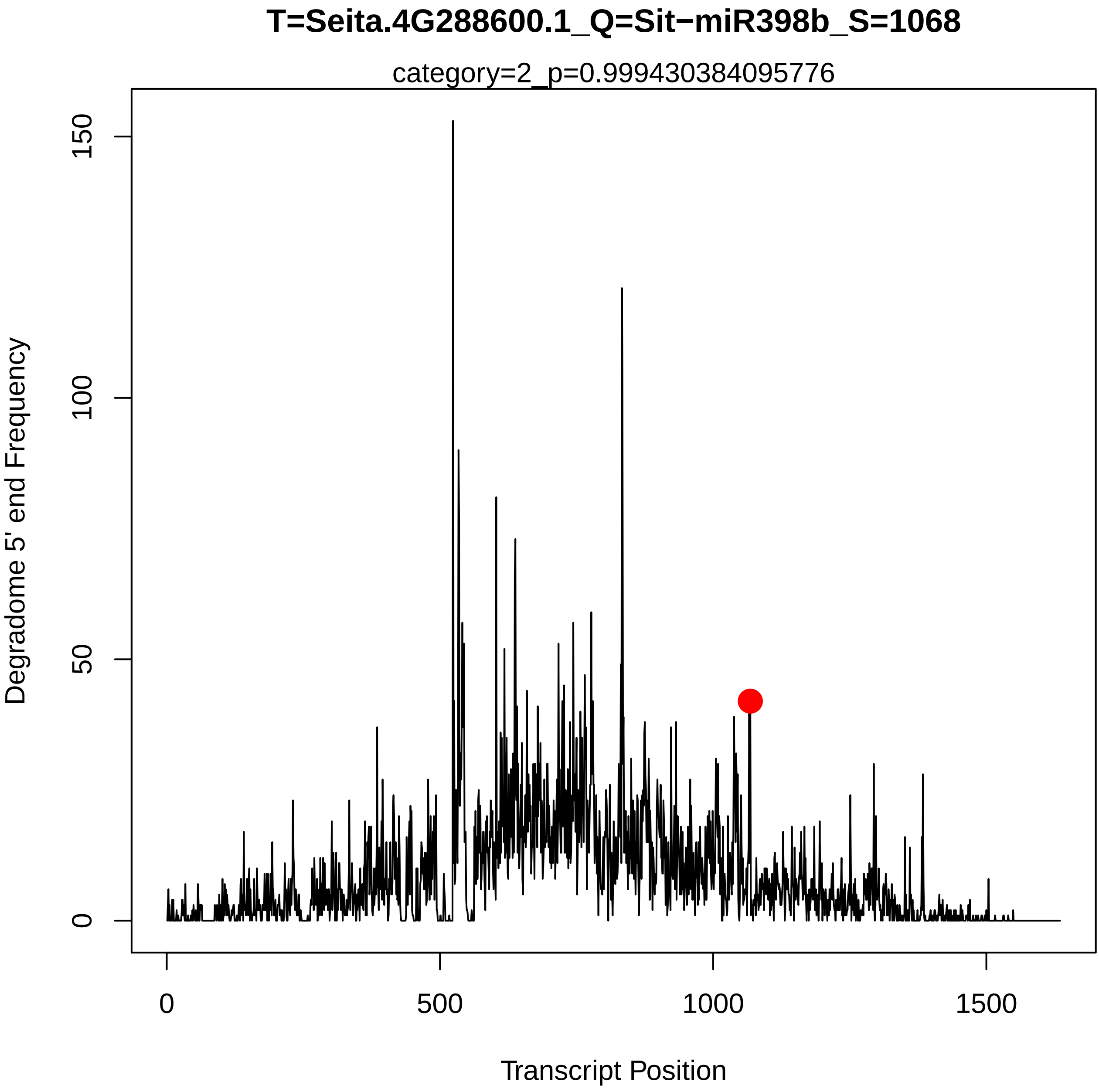

T=Seita.4G288600.1_Q=Sit−miR398b_S=1068
category=2_p=0.999430384095776
Degradome 5' end Frequency
0
50
100
150
0
500
1000
1500
Transcript Position
